# Idiopathic toe walking – are common podiatric treatment options based on evidence?

**DOI:** 10.1186/1757-1146-6-S1-O37

**Published:** 2013-05-31

**Authors:** Cylie Williams, Paul Tinley, Barry Rawicki

**Affiliations:** 1 Allied Health Research Unit, Southern Health, Cheltenham, VIC, 3192, Australia; 2 Department of Physiotherapy, Monash University, Frankston, VIC, 3199, Australia; 3 School of Podiatry, Charles Sturt University, Albury, NSW, 2460, Australia; 4 Victorian Paediatric Rehabilitation Service, Monash Childrens, Clayton, VIC 3680, Australia

## 

Idiopathic toe walking (ITW) is a condition that commonly presents to podiatrists. This presentation aims to give an overview of the quality of literature focused on the treatment of ITW and determine if common podiatric treatment modalities are evidence based. 24 articles reporting treatment modalities were appraised against the National Health and Medical Research Council Levels of Evidence. There currently is no evidence of any single treatment option having long term effect on ITW gait. The highest level of evidence was in support of serial casting or surgery with some evidence supporting the use of Botulism toxin Type A.

Footwear and orthotic intervention with or without stretching programs are reported treatments with no rigorous studies to support these modalities yet anecdotally these are reported effective. This article aims update the knowledge of podiatrists, to enhance how children who present with this gait style can be managed and highlight areas for future research.

